# Relationship between free fatty acids and diabetic retinopathy in male patients with type 2 diabetes

**DOI:** 10.1038/s41598-025-07001-w

**Published:** 2025-07-01

**Authors:** Jintao Chen, Yajing Huang, Chuanfeng Liu, Jingwei Chi, Lili Xu, Yangang Wang

**Affiliations:** Department of Endocrinology, The Affiliated Hospital of Qingdao University, Qingdao, 266003 China

**Keywords:** Diabetes, Dyslipidaemias

## Abstract

This study aims to evaluate the association between fasting serum free fatty acids (FFAs) and diabetic retinopathy (DR) in a population of Chinese men with type 2 diabetes mellitus (T2DM). A total of 781 eligible subjects participated in this cross-sectional study. We collected clinical data and information about the patient’s examination and laboratory tests. The Shapiro–Wilk test was employed to assess the normality of continuous variables. For continuous variables with non-normal distributions, the Mann–Whitney U test and the Kruskal-Wallis test was utilized for comparisons. Categorical variable comparisons were performed using the chi-square test. Logistic regression analyses and restricted cubic spline (RCS) curves were conducted to examine the association between FFAs levels and DR. FFAs levels were slightly lower in the DR group compared to the NDR group (0.35 vs. 0.41 mmol/L, *p* < 0.05). The logistic regression model did not identify a statistically significant linear relationship between FFAs levels and DR incidence (OR = 0.776, 95% CI: 0.308–1.955, *p* = 0.591). After adjusting for various confounding factors, RCS curves revealed a U-shaped relationship between FFAs and DR. This relationship was validated in subgroup analyses stratified by age, diabetes duration, BMI, and alcohol consumption status. The U-shaped curve was evident in most subgroups, indicating that both low and high levels of FFAs are associated with an increased risk of DR. IIn this study, we found a U-shaped relationship between FFAs and DR in Chinese men with T2DM. Both low and high levels of FFAs were associated with an increased risk of DR.

## Introduction

Diabetes mellitus is a chronic metabolic disease with rapidly increasing prevalence worldwide, imposing a significant burden on individuals and society. Diabetic retinopathy (DR) is one of the most common microvascular complications of type 2 diabetes mellitus (T2DM) and is a leading cause of blindness^1^. The global prevalence of DR has been estimated at around 35% among people with diabetes^2^. DR not only decreases the quality of life for patients but also results in substantial economic burdens^1,3^. Despite extensive research, the etiology of DR remains unclear. Therefore, evaluating other factors associated with DR is crucial for a better understanding of its occurrence and progression.

Free fatty acids (FFAs) are intermediates in lipid mobilization, primarily generated from lipolysis and triglyceride (TG) breakdown^4^. FFAs are carboxylic acids with long saturated or unsaturated chains. Most naturally occurring FFAs consist of a straight chain with 4–28 carbon atoms^5^. Beyond their role as energy sources, FFAs play critical roles in receptor signaling, gene expression, and systemic energy homeostasis under various physiological conditions^6,7^. However, excessive FFAs can induce endothelial dysfunction through various mechanisms, with elevated plasma FFAs levels significantly contributing to arterial dysfunction. FFAs mediate the regulation of several arterial functions, including endothelial dysfunction, angiogenesis, hypertension, and atherosclerosis^8–11^.

The relationship between FFAs and DR has not been thoroughly investigated. In this study, we analyzed the association between DR and FFAs in hospitalized male patients with T2DM.

## Methods

### Study population

This study included 781 male patients aged 30–80 years old with T2DM who were treated at the Endocrinology Department of Qingdao University Affiliated Hospital between 2016 and 2022. Patients with acute T2DM complications, severe heart failure, significant liver disease, renal insufficiency requiring dialysis, malignant tumors, or those using lipid-lowering medications were excluded from the study. The study protocol was approved by the Ethics Committee of Qingdao University Medical College Affiliated Hospital (Ethics Approval Number: [QYFY WZLL 25757]). As this study was retrospective and the data came from the affiliated hospital of Qingdao University, the Ethics Committee waived patient consent. Clinical trial registration number: ChiCTR2000033114. This research adhered to the STROBE guidelines and the Declaration of Helsinki principles.

### Data collection

Data collected for the study included age, height, weight, diabetes duration, alcohol and smoking status, blood pressure, fasting blood glucose (FBG), glycated hemoglobin (HbA1c), fasting C-peptide, fasting insulin, FFAs, low-density lipoprotein cholesterol (LDL), high-density lipoprotein cholesterol (HDL), TG, total cholesterol (TC), serum creatinine (SCr), serum uric acid (UA), alanine aminotransferase (ALT), aspartate aminotransferase (AST), and estimated glomerular filtration rate (eGFR), which was calculated using the CKD-EPI formula. All participants underwent DR assessment using a fundus camera (AFC-330; NIDEK, Kyoto, Japan), slit-lamp microscope (3020 H; Keeler Ltd, Windsor, UK), and non-invasive optical coherence tomography system (5000; Carl Zeiss, Dublin, CA, USA). Patients were diagnosed with DR based on multiple characteristic manifestations: (1) non-proliferative diabetic retinopathy: microaneurysm, hard exudates, cotton-wool spot and so on; (2) proliferative diabetic retinopathy: the formation of neovascularization which can lead to retinal detachment severely. Patients were grouped based on FFA levels into four quartiles: Q1: FFAs < 0.28 mmol/L, Q2: 0.28 ≤ FFAs < 0.4 mmol/L, Q3: 0.4 ≤ FFAs < 0.53 mmol/L, and Q4: FFAs ≥ 0.53 mmol/L.

### Statistical analysis

Statistical analyses were performed using R version 4.3.3. Shapiro-Wilk was used to test the normality of continuous variables. Normally distributed variables were expressed as the mean ± standard deviation, non-normal variables were expressed as the median (interquartile range), while categorical variables were expressed as the percentage (%). Group comparisons were conducted using the Chi-square test or Kruskal-Wallis test, as appropriate. Grouping based on quartiles of FFAs identifies confounders that require correction. Logistic regression analysis was used to examine the association between FFA levels and DR. Additionally, a restricted cubic spline (RCS) curve was utilized to explore the potential non-linear relationship between FFA and DR. Three nodes (knots) were set up based on the method proposed by Harrell, with node positions at the 10th, 50th, and 90th percentiles. A p-value < 0.05 was considered statistically significant.

## Results

### Baseline characteristics stratified by DR

This study included 781 male patients with T2DM, all of whom were not on lipid-lowering therapy. Patients were divided into two groups based on the presence of DR: the DR group (*n* = 178) and the non-DR group (NDR, *n* = 603). The Shapiro-Wilk test demonstrated non-normal distributions across all continuous variable. The baseline characteristics of the two groups are presented in Table 1. The analysis revealed that FFAs levels were slightly lower in the DR group compared to the NDR group (0.35 vs. 0.41 mmol/L, *p* = 0.034), suggesting a potential association between FFAs levels and the development of DR. Additionally, the DR group had a longer diabetes duration (10 vs. 8 years, *p* = 0.002) and higher HbA1c levels (8.6% vs. 7.9%, *p* < 0.001) than the NDR group, which are factors closely related to the onset and progression of retinopathy. Furthermore, fasting C-peptide and fasting insulin levels were significantly lower in the DR group, indicating potentially more severe pancreatic β-cell dysfunction. Notably, although the difference was small, the body mass index (BMI) was slightly lower in the DR group compared to the NDR group (25.6 vs. 26.0 kg/m², *p* = 0.046). There were no significant differences between the two groups in terms of age, smoking history, alcohol consumption, hypertension history, FBG, eGFR, LDL, HDL, TC, TG, and UA.

**Table 1:** Baseline information by diabetic retinopathy Status.

Characteristic	Diabetic retinopathy status		p-value
	NDR *N* = 603	DR *N* = 178	
Age(years)	58.00 (51.00, 64.00)	58.00 (52.00, 64.00)	0.842
Duration (years)	8.00 (3.00, 14.00)	10.00 (5.00, 16.00)	0.002
BMI (kg/m^2^)	26.00 (23.80, 28.50)	25.60 (23.20, 27.70)	0.046
Smoke			0.267
0	267 (45%)	69 (39%)	
1	332 (55%)	109 (61%)	
Drink			0.882
0	279 (47%)	86 (49%)	
1	321 (53%)	91 (51%)	
Hypertension			0.003
0	263 (44%)	100 (56%)	
1	340 (56%)	78 (44%)	
SBP (mmHg)	139.00 (126.00, 151.00)	140.00 (129.00, 154.00)	0.115
DBP (mmHg)	82.00 (75.00, 91.00)	83.00 (76.00, 91.00)	0.443
HbA1c (%)	7.90 (6.80, 9.40)	8.60 (7.20, 10.50)	< 0.001
FBG (mmol/L)	6.67 (5.48, 8.24)	6.87 (5.48, 8.93)	0.462
C-P (ng/mL)	2.02 (1.31, 2.86)	1.59 (0.95, 2.49)	< 0.001
INS (µIU/mL)	7.18 (4.20, 11.72)	5.77 (3.05, 11.24)	0.012
EGFR (mL/min/1.73 m²)	94.64 (82.54, 103.79)	95.28 (81.96, 102.77)	0.976
FFA (mmol/L)	0.41 (0.29, 0.53)	0.35 (0.23, 0.54)	0.034
HDL (mmol/L)	1.09 (0.92, 1.25)	1.10 (0.93, 1.34)	0.164
LDL (mmol/L)	2.70 (2.05, 3.21)	2.71 (1.90, 3.30)	0.866
TG (mmol/L)	1.40 (0.98, 2.36)	1.36 (0.94, 2.12)	0.274
TC (mmol/L)	4.48 (3.73, 5.18)	4.52 (3.60, 5.25)	0.726
UA (umol/L)	346.00 (280.00, 403.00)	332.00 (284.00, 398.00)	0.680
AST (U/L)	17.00 (14.00, 22.00)	17.00 (14.00, 21.00)	0.595
ALT (U/L)	19.00 (14.00, 29.00)	18.00 (14.00, 26.00)	0.070

Non-normal variables are expressed as the median (interquartile range) and categorical variables are expressed as the count (percentage). Comparisons between groups were made using the Mann-Whitney U test or chi-square test.

### Baseline characteristics stratified by FFAs quartiles

Based on FFA quartiles, all patients were divided into four groups, with baseline characteristics detailed in Table 2. As FFAs levels increased, the prevalence of DR initially increased and then decreased, suggesting a potential nonlinear relationship between FFAs and DR. This trend was further validated through RCS regression analysis. Additionally, with higher FFAs levels, there was a significant increase in BMI and a corresponding rise in the prevalence of hypertension. Moreover, FBG, fasting C-peptide, fasting insulin, and TC also showed a progressive upward trend, indicating that FFAs may be closely linked to various factors associated with metabolic syndrome. Conversely, the duration of diabetes tended to shorten as FFAs levels increased, which may reflect varying associations between FFAs levels and disease progression at different stages of diabetes. However, it is noteworthy that there were no significant differences in age, smoking history, alcohol consumption, HbA1c, eGFR, LDL, HDL, and TG across the different FFAs quartiles, suggesting that these factors may not play a dominant role in the variations of FFAs levels.

**Table 2:** Baseline characteristics by FFA quartiles.

Characteristic	FFA quartile				p-value
	1 *N* = 196	2 *N* = 195	3 *N* = 195	4 *N* = 195	
DR					< 0.001
NDR	130 (66%)	159 (82%)	165 (85%)	149 (76%)	
DR	66 (34%)	36 (18%)	30 (15%)	46 (24%)	
BMI (kg/m^2^)	24.80 (23.00, 26.20)	25.75 (23.70, 28.40)	26.30 (24.45, 28.70)	26.85 (24.40, 29.75)	< 0.001
Age (years)	59.00 (53.00, 64.00)	58.00 (51.00, 63.00)	57.00 (50.00, 63.00)	57.00 (49.00, 64.00)	0.446
Duration (years)	10.00 (3.00, 15.00)	8.00 (3.00, 15.00)	8.50 (3.00, 13.00)	7.00 (3.00, 15.00)	0.240
Smoke					0.601
0	86 (44%)	87 (45%)	76 (39%)	87 (45%)	
1	108 (56%)	108 (55%)	118 (61%)	107 (55%)	
Drink					0.325
0	93 (48%)	93 (48%)	85 (44%)	95 (49%)	
1	100 (52%)	102 (52%)	110 (56%)	99 (51%)	
Hypertension					< 0.001
0	126 (64%)	70 (36%)	63 (32%)	104 (53%)	
1	70(36%)	125(64%)	132(68%)	91(47%)	
SBP (mmHg)	137.00 (123.00, 152.00)	142.00 (129.00, 153.00)	138.50 (127.00, 150.00)	142.00 (128.00, 153.00)	0.053
DBP (mmHg)	79.50 (72.50, 87.00)	82.00 (76.00, 91.00)	82.00 (77.00, 92.00)	85.00 (77.00, 91.00)	< 0.001
HbA1c (%)	8.40 (6.90, 10.10)	7.80 (6.70, 9.50)	7.90 (6.90, 9.40)	8.20 (6.90, 9.50)	0.161
FBG (mmol/L)	6.40 (5.04, 7.91)	6.57 (5.32, 7.99)	6.86 (5.76, 8.51)	7.31 (5.74, 8.94)	< 0.001
C-P (ng/mL)	1.52 (0.84, 2.34)	1.91 (1.31, 2.49)	2.11 (1.46, 3.04)	2.17 (1.46, 3.20)	< 0.001
INS (µIU/mL)	5.51 (3.08, 9.01)	6.45 (3.98, 10.80)	7.74 (4.21, 11.73)	8.78 (4.74, 15.40)	< 0.001
EGFR (mL/min/1.73 m²)	94.21 (81.10, 101.35)	95.59 (82.41, 102.67)	94.11 (82.22, 103.45)	95.98 (85.49, 106.57)	0.168
FFA (mmol/L)	0.21 (0.16, 0.25)	0.34 (0.31, 0.36)	0.47 (0.43, 0.50)	0.66 (0.58, 0.76)	< 0.001
HDL (mmol/L)	1.09 (0.92, 1.31)	1.08 (0.94, 1.30)	1.07 (0.90, 1.21)	1.13 (0.93, 1.32)	0.112
LDL (mmol/L)	2.66 (2.04, 3.24)	2.70 (2.00, 3.19)	2.71 (2.07, 3.17)	2.72 (1.97, 3.40)	0.893
TG (mmol/L)	1.13 (0.83, 1.76)	1.36 (1.02, 1.94)	1.76 (1.09, 2.76)	1.67 (1.08, 3.21)	< 0.001
TC (mmol/L)	4.45 (3.58, 5.15)	4.46 (3.68, 5.15)	4.53 (3.75, 5.09)	4.64 (3.79, 5.47)	0.107
UA (umol/L)	319.00 (266.50, 381.50)	349.00 (276.00, 402.00)	353.00 (295.00, 410.00)	346.00 (299.00, 406.00)	0.019
AST (U/L)	16.00 (13.00, 19.00)	16.00 (14.00, 20.00)	18.00 (15.00, 23.00)	18.00 (16.00, 26.00)	< 0.001
ALT (U/L)	17.50 (13.50, 22.00)	17.00 (13.00, 25.00)	21.00 (15.00, 31.00)	23.00 (16.00, 37.00)	< 0.001

Non-normal variables are expressed as the median (interquartile range) and categorical variables are expressed as the count (percentage). Comparisons between groups were made using the Kruskal-Wallis test or chi-square test.

### Logistic regression analysis and RCS curve

In this study, we initially employed logistic regression analysis to investigate the association between FFAs and DR. The logistic regression model did not identify a statistically significant linear relationship between FFAs levels and DR incidence (OR = 0.776, 95% CI: 0.308–1.955, *p* = 0.591). Based on the results in Table 2 and in conjunction with the risk factors for DR, after adjusting for potential confounders, including age, diabetes duration, hypertension history, smoking status, alcohol consumption, BMI, HbA1c, fasting C-peptide, LDL, HDL, TC, and TG, the association between FFAs and DR remained non-significant (OR = 1.027, 95% CI: 0.380–2.777, *p* = 0.958) (Table 3).

**Table 3:** Relationship between FFAs and DR (Logistic regression).

	OR (95%CI)	*p*-value
Crued OR	0.776 (0.308, 1.955)	0.591
Mode 1	0.991 (0.383, 2.566)	0.985
Mode 2	1.065 (0.407, 2.786)	0.898
Mode 3	1.027 (0.380, 2.777)	0.958

Mode 1: Adjusted for age, duration, hypertension, BMI, history of drinking, history of smoking.

Mode 2: Adjusted for age, duration, hypertension, BMI, history of drinking, history of smoking, HbA1C, fasting C-peptide.

Mode 3: Adjusted for age, duration, hypertension, BMI, history of drinking, history of smoking, HbA1C, fasting C-peptide, LDL, HDL, TC, TG.

**Fig. 1:**
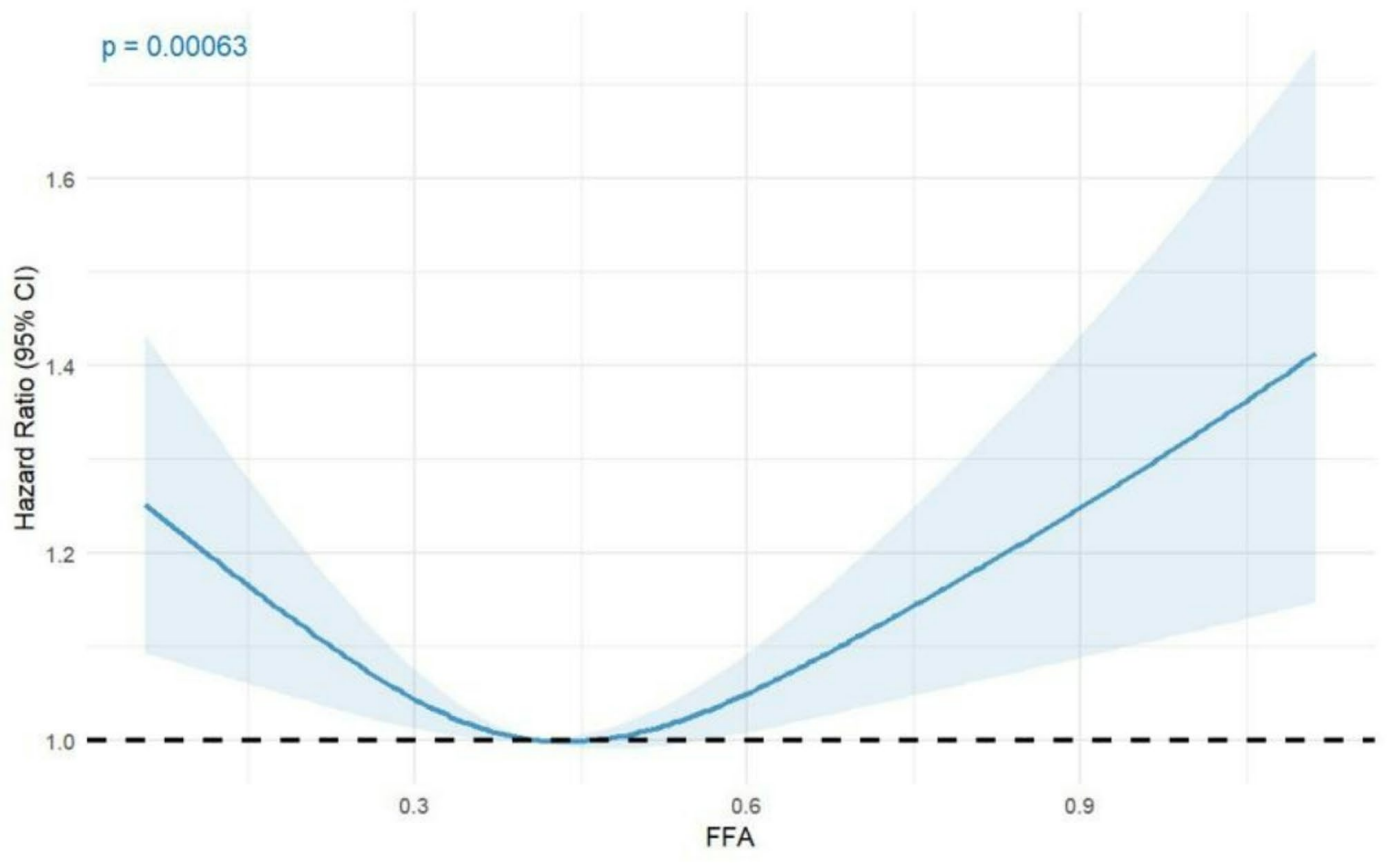
Non-liner relationship between FFAs and DR.

Subsequent analysis using RCS modeling identified a significant U-shaped association between FFAs levels and DR risk (p for nonlinearity = 0.0001; overall *p* = 0.00063) (Fig. 1). These findings highlight the necessity of accounting for the nonlinear relationship between FFAs and DR across the entire FFAs spectrum when evaluating its influence on DR.

### Subgroup analysis

**Fig. 2:**
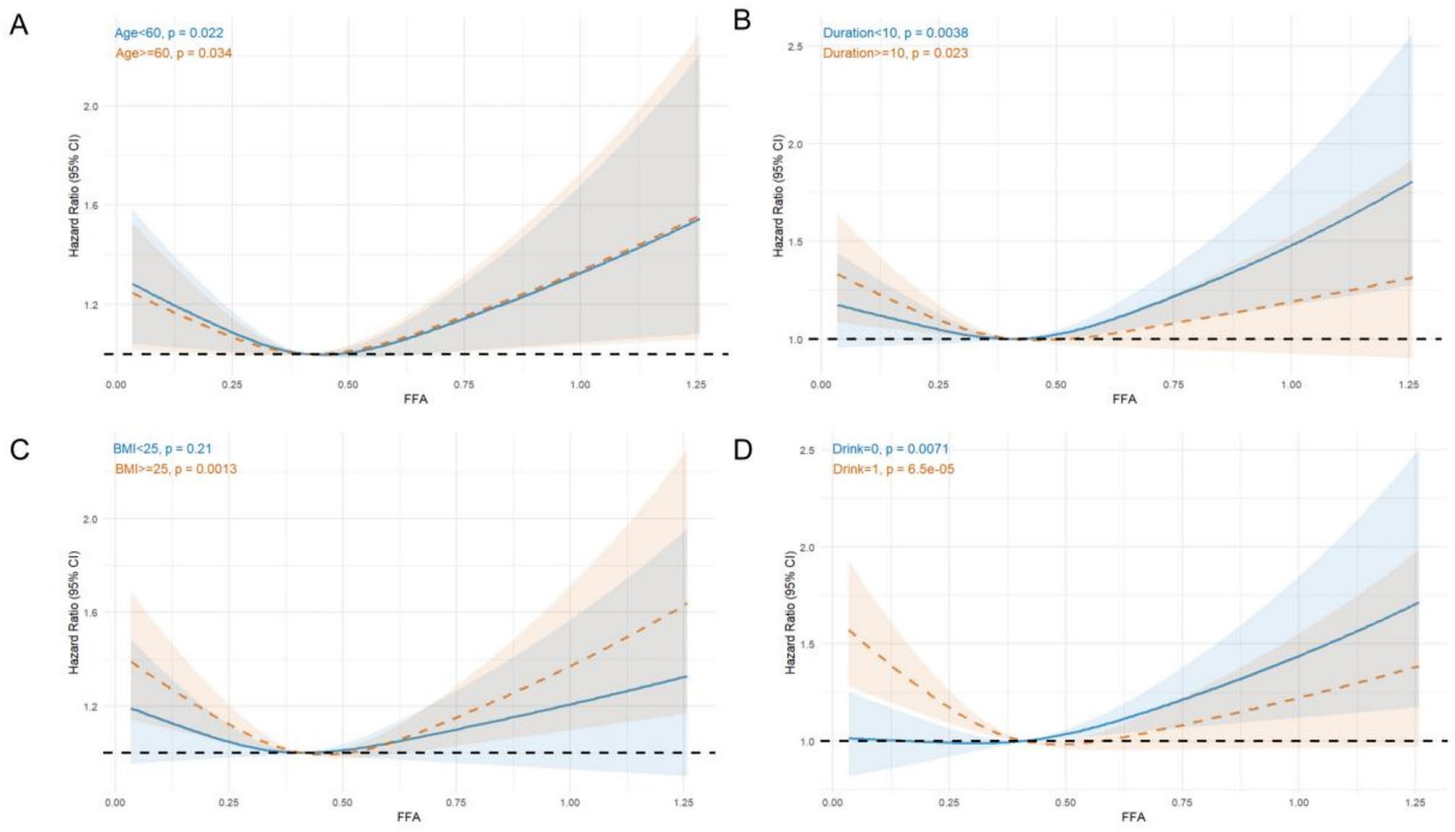
Non-liner relationship between FFAs and DR(Subgroup analysis).

Based on the subgroup analysis results from Fig. 2, a clear U-shaped relationship between FFAs levels and DR risk was observed in most subgroups. Specifically, in the age groups < 60 years vs. ≥60 years (Fig. 2A), diabetes duration groups < 10 years vs. ≥10 years (Fig. 2B), and drinking status groups non-drinkers vs. drinkers (Fig. 2D), a significant nonlinear relationship was evident, with DR risk initially decreasing and then increasing as FFAs levels rose, particularly at higher FFAs levels. However, in patients with a BMI < 25 (Fig. 2C), although the relationship did not reach statistical significance (*p* = 0.24), this result may be influenced by the limited sample size, and a similar U-shaped trend might still exist. Overall, these findings suggest that the relationship between FFAs levels and DR risk is potentially complex and nonlinear, with consistent trends across different subgroups, indicating that the role of FFAs in the development of DR warrants further investigation.

## Discussion

Our study revealed a significant U-shaped relationship between serum FFAs and DR, where both low and high levels of FFAs were associated with increased incidences of DR. This dual role of FFAs underscores the importance of maintaining optimal FFA slevels to prevent the progression of DR in diabetic patients. Notably, extremely low FFAs levels were also linked to a higher risk of DR. There is a strong correlation between dyslipidaemia and DR, and in the Fenofibrate Intervention in Diabetes and Event Lowering (FIELD) and Action to Control Cardiovascular Risk in Diabetes (ACCORD) studies the lipid-lowering drug fenofibrate delayed the progression of retinopathy independently of glycaemic control^12,13^. Serum levels of saturated fatty acids such as palmitic acid (PA) are significantly elevated in diabetic patients, and together with high glucose levels, PA promotes the expression of DR-associated angiogenic and inflammatory targets, including PTGS2 and CXCL8, in human Müller cells^14^. These pro-inflammatory cytokines are potent stimulators of retinal endothelial pathology, such as leukocyte stasis, vascular permeability and basement membrane thickening^15^. In addition, elevated PA promotes apoptosis of retinal microvascular pericytes (PCs) through pathways such as oxidative stress, ceramide synthesis, and NF-κB activation^16^.

Similarly, other studies have found that low FFAs levels are associated with poor clinical outcomes, such as in out-of-hospital cardiac arrest (OHCA) patients, where lower FFAs levels were significantly linked to worse neurological outcomes^17^ .These findings collectively suggest that FFAs may have a protective role under certain pathological conditions, while exacerbating disease progression under others. This may be because moderate FFAs serve as an essential energy source for retinal cells, helping to sustain their normal function^18^. We speculate that in diabetic patients, insulin insufficiency or resistance leads to reduced glucose uptake by cells, particularly during hypoglycemia, exacerbating DR^19^. At such times, FFAs may act as an alternative energy source, maintaining cellular function and mitigating damage caused by energy deficits, which might explain the negative correlation between FFAs and DR at certain concentrations.

Interestingly, prior studies have also explored the role of specific fatty acids in DR. For example, a study highlighted that higher levels of omega-3 fatty acid DHA were inversely associated with the presence and severity of diabetic retinopathy in patients with type 2 diabetes^20^. Similarly, a propensity score-matched case-control study highlighted a significant negative association between n-6 PUFA levels and DR^21^. These findings align with our results, suggesting that not only total FFAs but also specific PUFA subtypes, such as n-6 PUFAs, may play crucial roles in modulating DR risk. Future research could focus on differentiating the effects of various FFAs and PUFAs on DR, which may offer new therapeutic targets for preventing this complication in diabetic patients.

However, as FFAs levels continue to rise, their relationship with retinopathy gradually shifts to a positive correlation, a finding that aligns closely with the results of Lakshmi Kanta Mondal et al. Their study conducted an in-depth cross-sectional analysis of lipid profiles in patients with T2DM and DR, revealing that blood levels of FFAs were significantly elevated in patients with DR^22^. In contrast, our study not only included a larger sample size of male patients but also identified and established for the first time a U-shaped relationship between FFAs and DR. Although studies directly linking total FFAs to DR are relatively scarce, the detrimental effects of excessive fatty acids on metabolic systemic diseases have been well documented in the literature. For instance, research has clearly demonstrated that FFAs are significantly associated with insulin resistance^23^ and the development of T2DM^24^, and they also serve as an independent and potent risk factor for hypertension^25^. The potential mechanisms underlying the positive correlation between high FFAs levels and retinopathy may include several aspects: Firstly, FFAs can activate multiple inflammatory pathways, leading to a significant increase in the expression of inflammatory mediators such as tumor necrosis factor-α (TNF-α), monocyte chemoattractant protein-1 (MCP-1), and interleukin-6 (IL-6), and promoting the secretion of adhesion molecules such as ICAM-1 and VCAM-1, thereby initiating and exacerbating endothelial dysfunction^8,10,26,27^. Additionally, FFAs are a major source of reactive oxygen species (ROS); excessive FFAs can increase ROS production by activating pathways involving protein kinase C (PKC) and NADPH oxidase, thereby inducing oxidative stress^25,28,29^, which further damages tissues and cells. Moreover, high levels of FFAs may induce endothelial cell apoptosis and exacerbate endothelial damage through the protein phosphatase 2Cb (PP2Cb) and TLR4 signaling pathways^30,31^. In summary, this U-shaped relationship suggests that maintaining appropriate FFA levels may have significant clinical implications for the long-term management of diabetes, not only in delaying or preventing the onset of DR but also in improving overall metabolic outcomes and prognosis in patients.

This study has several limitations. First, as a cross-sectional study, we cannot infer causality. Second, all recruited patients were hospitalized in the same region, so the results may not be generalizable to other populations. Third, there is currently a lack of sufficient experimental evidence to explain the relationship between FFAs and DR.

In the future, we aim to explore new research areas regarding the relationship between FFAs and DR. The potential role of FFAs in the treatment of DR still needs to be carefully and comprehensively evaluated. Large-scale prospective cohort studies are necessary to determine the causal relationship between FFAs and DR. Furthermore, our current measurements are of total FFAs, without differentiating their specific types. Future research needs to clarify the specific relationships between different types of FFAs and DR.

## Conclusion

In this study, we found a U-shaped relationship between FFAs and DR in Chinese men with T2DM. Both low and high levels of FFAs were associated with an increased risk of DR. Future, more complex prospective studies are needed to confirm these findings and explore their causal relationships. This suggests that monitoring and managing FFA levels may be crucial in reducing the incidence of DR in T2DM patients.

## Data Availability

Clinical data for this study can be obtained by contacting the corresponding author.
